# Supplementary material for: Patterns of Intron Gain and Loss in Fungi
Source: PLoS Biol. 2004 Nov 30;2(12):e422. doi: 10.1371/journal.pbio.0020422 (PMC532390; doi:10.1371/journal.pbio.0020422)
Supplement: Table S1 — Also available at http://genes.mit.edu/NielsenEtAl/. (4.3 MB ZIP). [file pbio.0020422.st001.zip › NielsenEtAl/html/1244.html]

AN6510.1.NCU01179.1.MG08637.1.FG07331.1


```
 CLUSTAL W (1.82) Multiple Sequence Alignments - Introns Inserted


Sequence 1: MG08637.1	355 aa
Sequence 2: FG07331.1	356 aa
Sequence 3: NCU01179.1	349 aa
Sequence 4: AN6510.1	355 aa
Alignment Length: 365 aa
Number Identitical Residues: 126 aa
Alignment Score (without introns) 7634


MG08637.1 	MAS-ATLDIMR----NNPLTASVADVYSSFHERRAKLGLTNPGTVENLAKE~VQRDVFTN
NCU01179.1	MASFSTESPLAMLR-DNAIYSSLSDAFNAFQERRKQFGLSNPGTIETIARE1VQRDTLLT
FG07331.1 	MASTAALDPVKKALLSNPLAGALGDVFNSFNERRAKLGLSNPGTIESLAKE~VQRDVFLN
AN6510.1  	MADHLALPSFLT---DNSVVSALLDTYTSFSERRAALGLPNPGTVENVGRE~VQKDVLLS
          	**.  :   .     .*.: .:: *.:.:* ***  :**.****:*.:.:* **:*.: .

MG08637.1 	NYAFSGLRGDFTRAFNLNPL~FQVSHNFAVG--ERMPPYTFAAMYGSSK0VFMQGSVDND
NCU01179.1	NYMFSGLRADVTKAFSLAPL2FQVSHQFAMG--ERLNPYAFAALYGTNQ0IFAQGNLDNE
FG07331.1 	NYMFTGIRADLTKIFSMAPL~FQVSHQFAMG--ERINPYTFAAMYGTGK0VFCQGNLDNE
AN6510.1  	NFMFSGLRADLTKMFSMAPL~FRVSHAFSMGGSGNMAPYAFSAMYGTSS0VFMQGNFGSD
          	*: *:*:*.*.*: *.: ** *:*** *::*.: .: **:*:*:**:.. :* **....:

MG08637.1 	MQVNARFNWRWSDALVSKTQFSLGTGNQD-MAHLESEYTGSDFTTSIKALNPSFFEGGLT
NCU01179.1	GALSTRFNYRWGDRTITKTQFSIG-GGQD-MAQFEHEHLGDDFSASLKAINPSFLDGGLT
FG07331.1 	GSLSGRFNWRWSDKLVSKSQLSISPGGQD-MAQFEHEYTGNDFSASLKMLNPSYLEGGVT
AN6510.1  	GGLAALYNYRWTPKLVTKTNVQIMPGAEQGLIQLDNDYTGDDFSLSLKAFNPSYLDGGLT
          	  :   :*:**    ::*::..: .* ::.: ::: :: *.**: *:* :***:::**:*

MG08637.1 	GIFMGSYMQSVTKKLALGMEGVWQRAALSQPPDTVMSYVAKYKSDDWIATGQL-AGAGAL
NCU01179.1	GIFVGDYLQAVTPRLGLGLQAVWQRQGLTQGPDTAISYFARYKAGDWVASAQL-QAQGAL
FG07331.1 	GIFIGSYLQSVTSKLALGLETLWQRPSLSQGPECAVSYAARYKSADWIATAQLQAAMGTL
AN6510.1  	GIFVGSYLQSVTPKLALGFEAIWQRQGLNTRPESAVSYSARYKSDDWIASAQL-QAQGVF
          	***:*.*:*:** :*.**:: :*** .*.  *: .:** *:**: **:*:.**  . *.:

MG08637.1 	STTFWRKISEKCQAGIEMTLSLAPGDPAGGMMMGGPSIQAEGVTTAAVKYDFRMSTLRMQ
NCU01179.1	NTSFWKKLTDRVQAGVDMTLSVAP----SQSMMGG--LTKEGITTFGAKYDFRMSTFRAQ
FG07331.1 	NTSYWRRLSDKVQAGVDLSLGLVP--SPGGLMGGG--LQKEGVTTIGAKYDFRMSTFRAQ
AN6510.1  	TASYWKKISERVEAGVDMNLQFAP--NAAAMMMGG--PSKDGTTAIGAKYDFRASTFRAQ
          	.:::*:::::: :**:::.* ..*   ..  * **     :* *: ..***** **:* *

MG08637.1 	VDTKGKMTCLAEKRI-APAIMMTIAAEVDHPT0GAAKLGLGVSLESGGEELQERQEALGA
NCU01179.1	IDSKGKLSCLLEKRLGAAPVTLTFAADVDHVT0QQAKLGMSVSIEASDVDLQEQQE----
FG07331.1 	VDSKGKLSCALEKRV-APPVMMTFAADIDHFT0HQAKIGLGVSIEAAPEELQEQQEVLGA
AN6510.1  	VDSAGKVSCLLEKRI-AMPIALTFAGEIDQAK0QSAKVGLAVSLEIAGEEVMEQTEK-AD
          	:*: **::*  ***: * .: :*:*.::*: .   **:*:.**:* .  :: *: *  . 

MG08637.1 	AAPSPNIPF
NCU01179.1	GAQSLNIPF
FG07331.1 	QAP-PNIPF
AN6510.1  	PSTMVTPPF
          	 :   . **
```
